# Supplementary material for: KANK2 at focal adhesions regulates their maintenance and dynamics, while at fibrillar adhesions it influences cell migration via microtubule-dependent mechanism
Source: Cell Commun Signal. 2026 Mar 3;24:224. doi: 10.1186/s12964-026-02771-w (PMC13081611; doi:10.1186/s12964-026-02771-w)
Supplement: Supplementary file 1 — Supplementary Material 1. Supplementary Table S1. [file 12964_2026_2771_MOESM1_ESM.pdf]

Additional file 1

KANK2 at focal adhesion regulates their maintenance and dynamics, while at fibrillar adhesions it influences cell migration via microtubule-dependent mechanism

N. Stojanović[1,*,#, ORCID:0000-0002-7763-4154], A. Rac[1,*, ORCID:0000-0001-8821-3059], M. Lončarić[1, ORCID:0000-0002-5343-0368], A. Tadijan[1,2, ORCID:0000-0002-5487-3611], M. Paradžik[1,3, ORCID:0000-0003-1025-5595], M. Acman[1], J.D. Humphries[4, ORCID:0000-0002-8953-7079], M.J. Humphries[5, ORCID:0000-0002-4331-6967], A. Ambriović-Ristov[1,#, ORCID:0000-0001-7784-2466]

[1]Laboratory for Cell Biology and Signalling, Division of Molecular Biology, Ruđer Bošković Institute, Zagreb, Croatia; [2]present address: Laboratory for Cell Biology, Division of Molecular Biology, Ruđer Bošković Institute, Zagreb, Croatia; [3]present address: Laboratory of Experimental Therapy, Division of Molecular Medicine, Ruđer Bošković Institute, Zagreb, Croatia, [4]Department of Life Science, Manchester Metropolitan University, Manchester, United Kingdom; [5]Manchester Cell-Matrix Centre, Faculty of Biology, Medicine & Health, University of Manchester, Manchester, United Kingdom

*equal contribution

[#]corresponding authors, Nikolina.Stojanovic@irb.hr , Andreja.Ambriovic.Ristov@irb.hr

Table S1

Supplementary Table S1. List of used antibodies and dyes.

**WESTERN BLOT**					
Primary antibodies	***Ref. No.***	***Distributor***	***Monoclonal/polyclonal***	***Species***	***Dilution***
Anti-Liprin β1	sc-514575	Santa Cruz Biotechnology, USA	Monoclonal	Mouse	1:100 in 5% milk
Anti-FAK antibody [EP695Y]	ab40794	Abcam, USA	Monoclonal	Rabbit	1:1000 in 5% milk
Anti-Paxillin [Y113]	ab32084	Abcam, USA	Monoclonal	Rabbit	1:7500 in 5% milk
Anti-EEA1	2411	Cell Signaling Technology, USA	Polyclonal	Rabbit	1:1000 in 5% milk
Anti-LDH	sc33781	Santa Cruz Biotechnology, USA	Polyclonal	Rabbit	1:400 in 5% milk
Anti-plectin (10F6)	sc-33649	Santa Cruz Biotechnology, USA	Monoclonal	Mouse	1:200 in 5% milk
Anti-human talin2	MCA4771GA	Bio-Rad, USA	Monoclonal	Mouse	1:1000 in 5% milk
Anti-IQGAP1	ab133490	Abcam, USA	Monoclonal	Rabbit	1:1000 in 5% milk
Anti-vinculin	ab129002	Abcam, USA	Monoclonal	Rabbit	1:1000 in 5% milk
Anti-α-actinin 1(H-2)	sc-17829	Santa Cruz Biotechnology, USA	Monoclonal	Mouse	1:500 in 5% milk
Anti-α-actinin-4 (G-4)	sc-390205	Santa Cruz Biotechnology, USA	Monoclonal	Mouse	1:250 in 5% milk
Anti-Integrin α5	MAB18642	Bio-techne, USA	Monoclonal	Mouse	1:500 in 5% milk
Anti-Integrin β5	D24A5	Cell Signaling Technology, USA	Monoclonal	Mouse	1:1000 in 5% milk
Anti-zyxin	sc-136128	Santa Cruz Biotechnology, USA	Monoclonal	Mouse	1:500 in 5% milk
Anti-KANK2	HPA015643	Sigma-Aldrich, USA	Polyclonal	Rabbit	1:1000 in 5% milk
Secondary antibodies	***Ref. No.***	***Distributor***	***Monoclonal/ polyclonal***	***Species***	***Dilution***
Goat anti-rabbit IgG (H+L)	31466	Invitrogen, USA	Polyclonal	Goat	1:5000 in 5% milk
Goat anti-mouse IgG (H+L)	G21040	Invitrogen, USA	Polyclonal	Goat	1:10 000 in 5% milk
**FLOW CYTOMETRY**					
Primary antibodies	***Ref. No.***	***Distributor***	***Monoclonal/polyclonal***	***Species***	***Dilution***
Mouse IgG1 Negative Control	MABC002	Merck Millipore, Germany	Monoclonal	Mouse	1:50
Anti-Integrin αV	407286	Merck Millipore, Germany	Monoclonal	Mouse	1:100
Anti-Integrin beta 1	MAB1965	Merck Millipore, Germany	Monoclonal	Mouse	1:100
Secondary antibody	***Ref. No.***	***Distributor***	***Monoclonal/ polyclonal***	***Species***	***Dilution***
FITC Goat anti-Mouse Ig	554001	BD Pharmingen, USA	Polyclonal	goat	1:50

IMMUNOFLUORESCENCE					
Primary antibodies	***Ref. No.***	***Distributor***	***Monoclonal/ polyclonal***	***Species***	***Dilution***
Anti-human talin1	MCA4770GA	Bio-Rad, USA	Monoclonal	Mouse	1:100 in 5% BSA
Anti-human talin2	MCA4771GA	Bio-Rad, USA	Monoclonal	Mouse	1:100 in 5% BSA
Anti-Integrin αV	MABT207	Merck Millipore, Germany	Monoclonal	Mouse	1:50 in 5% BSA
Anti-Integrin β5	D24A5	Cell Signaling Technology, USA	Monoclonal	Mouse	1:600 in 5% BSA
Anti-KANK2	HPA015643	Sigma-Aldrich, USA	Polyclonal	Rabbit	1:100 in 5% BSA
Anti-Integrin α5	NBP2-50146	Novus Biologicals, USA	Monoclonal	Mouse	1:500 in 5% BSA
Anti-alpha tubulin	CP06	Sigma-Aldrich, USA	Monoclonal	Mouse	1:20 in 5% BSA
Anti-Filamin 1 (E-3)	sc-17749	Santa Cruz Biotechnology, USA	Monoclonal	Mouse	1:100 in 5% BSA
Anti-Filamin B	ab97457	Abcam, USA	Polyclonal	Rabbit	1:50 in 5% BSA
Anti-α-actinin 1(H-2)	sc-17829	Santa Cruz Biotechnology, USA	Monoclonal	Mouse	1:50 in 5% BSA
Anti-α-actinin-4 (G-4)	sc-390205	Santa Cruz Biotechnology, USA	Monoclonal	Mouse	1:50 in 5% BSA
Anti-vinculin	ab129002	Abcam, USA	Monoclonal	Rabbit	1:100 in 5% BSA
Recombinant Alexa Fluor® 647 Anti-Vinculin	ab196579	Abcam, UK	Monoclonal	Rabbit	1:200 in 5% BSA
Secondary antibodies	***Ref. No.***	***Distributor***	***Monoclonal/polyclonal***	***Species***	***Dilution***
Anti-Mouse IgG Alexa Fluor 546	A-11030	Invitrogen, USA	Polyclonal	Goat	1:1000 in 5% BSA
Anti-Mouse IgG Alexa Fluor 488	#4408	Cell Signaling Technology, USA		Goat	1:1000 in 5% BSA
Anti-Mouse IgG Alexa Fluor 405	A-31553	Invitrogen, USA	Polyclonal	Goat	1:250 in 5% BSA
Anti-Rabbit IgG Alexa Flour, 555	A-31572	Invitrogen, USA	Polyclonal	Donkey	1:1000 in 5% BSA
Anti-Rabbit IgG Alexa Fluor 647	#4414	Cell Signaling Technology, USA	Polyclonal	Goat	1:1000 in 5% BSA
Anti-Mouse IgG1 Alexa Fluor 555	A-21127	Invitrogen, USA	Polyclonal	Goat	1:1000 in 5% BSA
Anti-Mouse $IgG2_b$ Alexa Fluor 488	A-21141	Invitrogen, USA	Polyclonal	Goat	1:1000 in 5% BSA
Dyes	***Ref. No.***	***Distributor***			***Dilution***
Phalloidin, Alexa Fluor 488	P5282	Sigma Aldrich, USA			1:100 in 5% BSA
**PROXIMITY LIGATION ASSAY (PLA)**					
Primary antibodies	***Ref. No.***	***Distributor***	***Monoclonal/polyclonal***	***Species***	***Dilution***
Anti-Integrin β5	D24A5	Cell Signaling Technology, USA	Monoclonal	Mouse	1:4000 in diluent*
Anti-human talin2	MCA4771GA	Bio-Rad, USA	Monoclonal	Mouse	1:4000 in diluent*

Anti-human talin1	MCA4770GA	Bio-Rad, USA	Monoclonal	Mouse	1:4000 in diluent*
Anti-KANK2	HPA015643	Sigma-Aldrich, USA	Polyclonal	Rabbit	1:4000 in diluent*
Anti-Integrin α5	NBP2-50146	Novus Biologicals, USA	Monoclonal	Mouse	1:4000 in diluent*
Secondary antibodies	***Ref. No.***	***Distributor***	***Monoclonal/ polyclonal***	***Species***	***Dilution***
Navenibody M1 (40X)	NB.1.100.06	Navinci Diagnostics AB, Sweden		Mouse	1:40 in diluent*
Navenibody R2 (40X)	NB.1.100.07	Navinci Diagnostics AB, Sweden		Rabbit	1:40 in diluent*

** part of NaveniFlexTM Cell MR kit*
