# Supplementary material for: KANK2 at focal adhesions regulates their maintenance and dynamics, while at fibrillar adhesions it influences cell migration via microtubule-dependent mechanism
Source: Cell Commun Signal. 2026 Mar 3;24:224. doi: 10.1186/s12964-026-02771-w (PMC13081611; doi:10.1186/s12964-026-02771-w)
Supplement: Supplementary file 3 — Supplementary Material 3. Supplementary Fig S9-S13. [file 12964_2026_2771_MOESM3_ESM.pdf]

Additional file 2

KANK2 at focal adhesion regulates their maintenance and dynamics, while at fibrillar adhesions it influences cell migration via microtubule-dependent mechanism

N. Stojanović[1,*,#, ORCID:0000-0002-7763-4154], A. Rac[1,*, ORCID:0000-0001-8821-3059], M. Lončarić[1, ORCID:0000-0002-5343-0368], A. Tadijan[1,2, ORCID:0000-0002-5487-3611], M. Paradžik[1,3, ORCID:0000-0003-1025-5595], M. Acman[1], J.D. Humphries[4, ORCID:0000-0002-8953-7079], M.J. Humphries[5, ORCID:0000-0002-4331-6967], A. Ambriović-Ristov[1,#, ORCID:0000-0001-7784-2466]

[1]Laboratory for Cell Biology and Signalling, Division of Molecular Biology, Ruđer Bošković Institute, Zagreb, Croatia; [2]present address: Laboratory for Cell Biology, Division of Molecular Biology, Ruđer Bošković Institute, Zagreb, Croatia; [3]present address: Laboratory of Experimental Therapy, Division of Molecular Medicine, Ruđer Bošković Institute, Zagreb, Croatia, [4]Department of Life Science, Manchester Metropolitan University, Manchester, United Kingdom; [5]Manchester Cell-Matrix Centre, Faculty of Biology, Medicine & Health, University of Manchester, Manchester, United Kingdom

*equal contribution

#corresponding authors, Nikolina.Stojanovic@irb.hr , Andreja.Ambriovic.Ristov@irb.hr

Supplementary Methods

SDS-PAGE and Western Blotting (related to Supplementary Fig. S1, S2B,C,D and S5)

Total cell lysates were obtained from 3.5 cm Petri dishes in 200 µL RIPA buffer supplemented with protease inhibitor cocktail (ThermoFisher). Samples for SDS-PAGE were collected by scraping. Samples containing an equal amount of protein were mixed in 6× Laemmli loading buffer (375 mM Tris-HCl (pH 6.8), 30% (w/v) glycerol, 12% (w/v) SDS, 0.02% (w/v) bromophenol blue, 12% (v/v) 2-mercaptoethanol) to reach a final 1× concentration, sonicated and heated for 5 min at 96°C. Isolated IACs were prepared for SDS-PAGE by solubilization in 2× Laemmli loading buffer and heating for 20 min at 70°C while shaking (1000 rpm). All samples were loaded onto pre-casted gradient gel (4 – 15% Mini-PROTEAN TGX) (Bio-Rad), separated by SDS-PAGE and semi-dry transferred to nitrocellulose (Bio-Rad). The membrane was blocked in 5% (w/v) non-fat dry milk or 5% (w/v) bovine serum albumin (BSA, Carl Roth), and incubated overnight with the appropriate antibodies, followed by incubation with horseradish peroxidase coupled secondary antibody. The primary and secondary antibodies are listed in Additional File 1, Supplementary Table S1. Detection was performed using chemiluminescence (PerkinElmer) and documented with Uvitec Alliance Q9 mini (BioSPX b.v.). Blots were quantified using ImageJ.

Flow cytometry (related to Supplementary Fig. S2A)

Adherent cells were grown in tissue culture dishes, detached by EDTA (Invitrogen, United States) and washed twice with PBS. Membrane fluorescence staining was performed using unlabelled primary antibodies (1 h, 4 C) for integrin subunit- aV or b1, while its binding was revealed by incubation (30 min, 4 C) of FITC-conjugated anti-mouse antibody. Isotype control samples were incubated with mouse IgG1 followed by FITC-conjugated anti-mouse antibody. Flow cytometry experiments were performed using FACSCalibur, while cell acquisition was made using BD CellQuest software package (all BD Biosciences, United States). Data were analysed using FCS Express 3 (De Novo Software, United States) software. All antibodies are listed in Additional File 1, Supplementary Table S1.

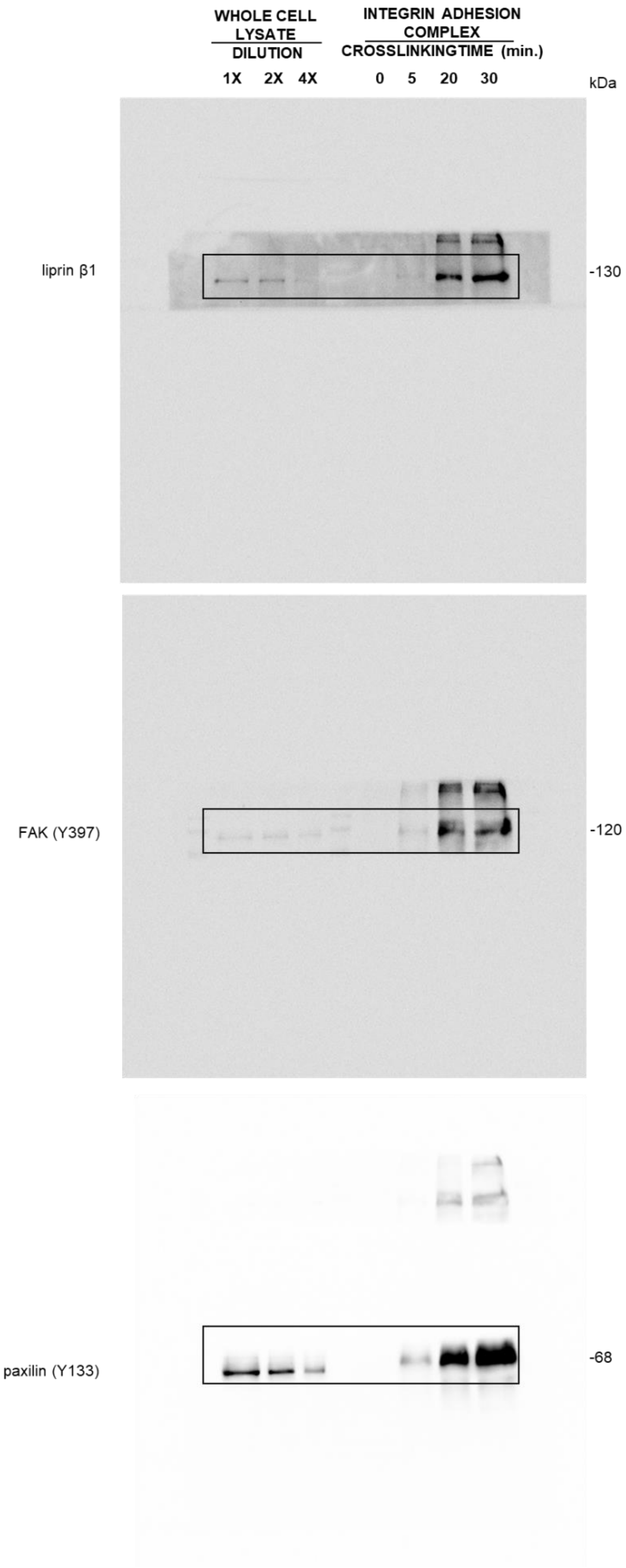

WHOLE CELL LYSATE
DILUTION
INTEGRIN ADHESION COMPLEX
CROSSLINKINGTIME (min.)
1X 2X 4X
0 5 20 30
kDa
liprin β1
-130
FAK (Y397)
-120
paxilin (Y133)
-68

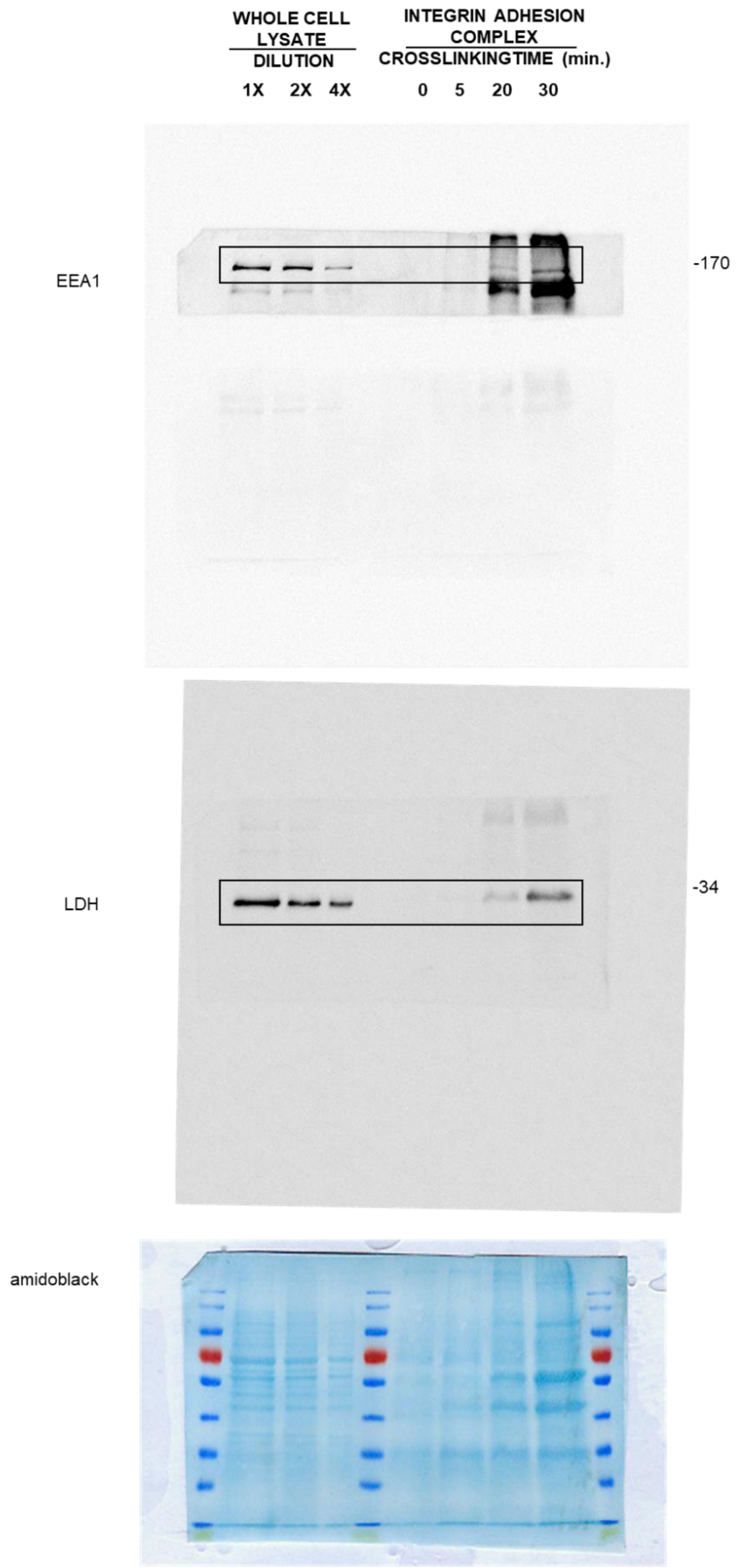

Supplementary Fig. S9 Full images of the blots in Fig. S1. Images were obtained using Uvitec Alliance Q9 mini, which directly scanned membranes developed with ECL reagents.

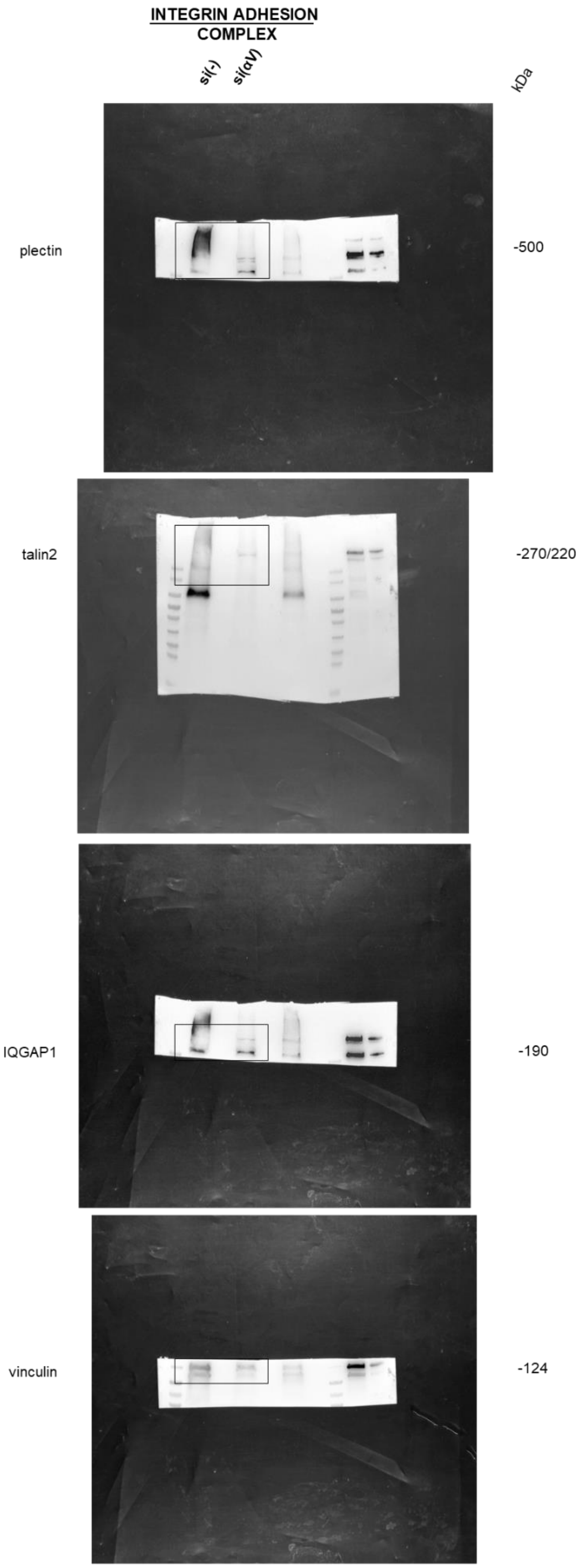
INTEGRIN ADHESION
COMPLEX
si(-)
si(αV)
kDa
plectin
-500
talin2
-270/220
IQGAP1
-190
vinculin
-124

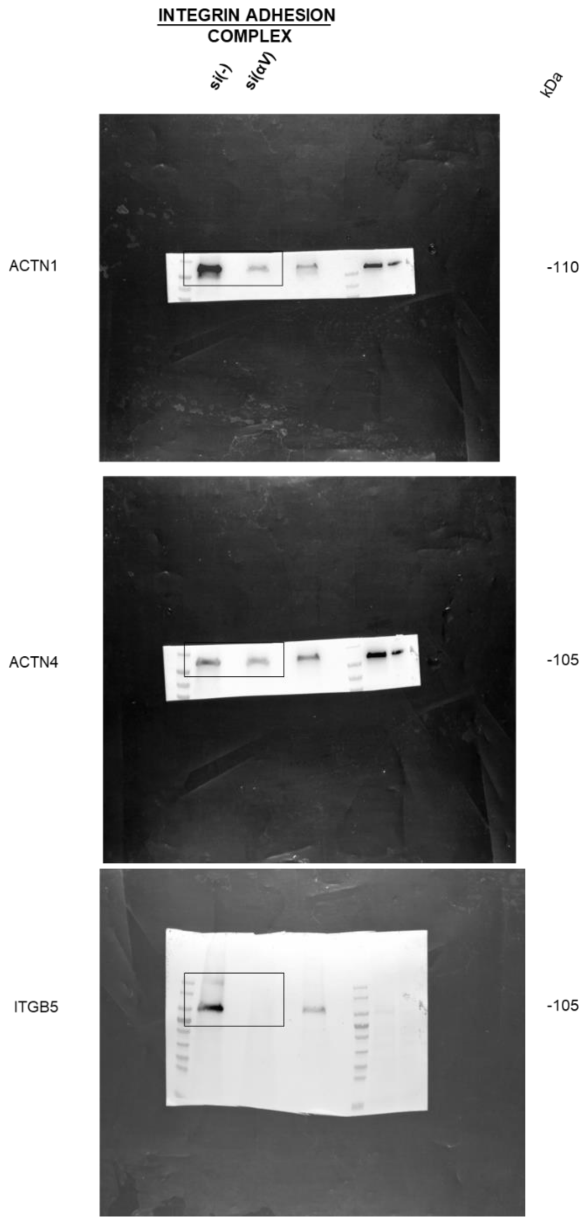

INTEGRIN ADHESION COMPLEX
si(-)
si(αV)
kDa
ACTN1
-110
ACTN4
-105
ITGB5
-105

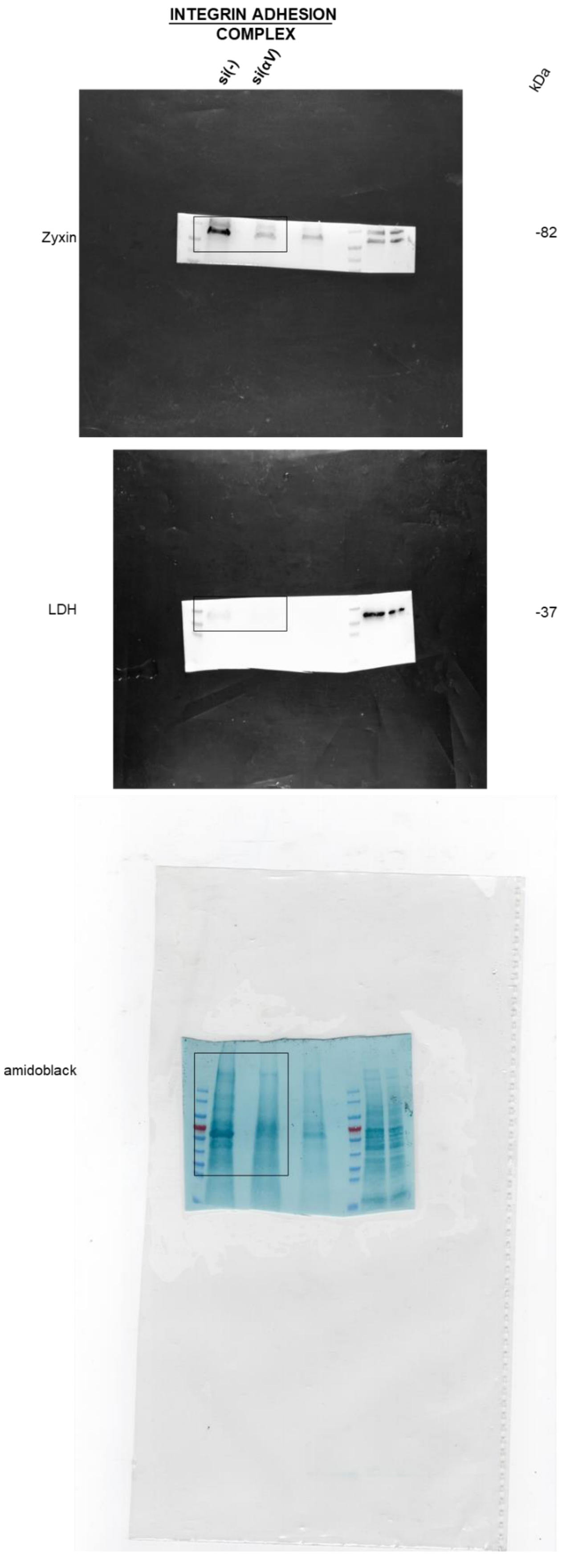

Supplementary Fig. S10 Full images of the blots in Fig. S2B. Images were obtained using Uvitec Alliance Q9 mini, which directly scanned membranes developed with ECL reagents.

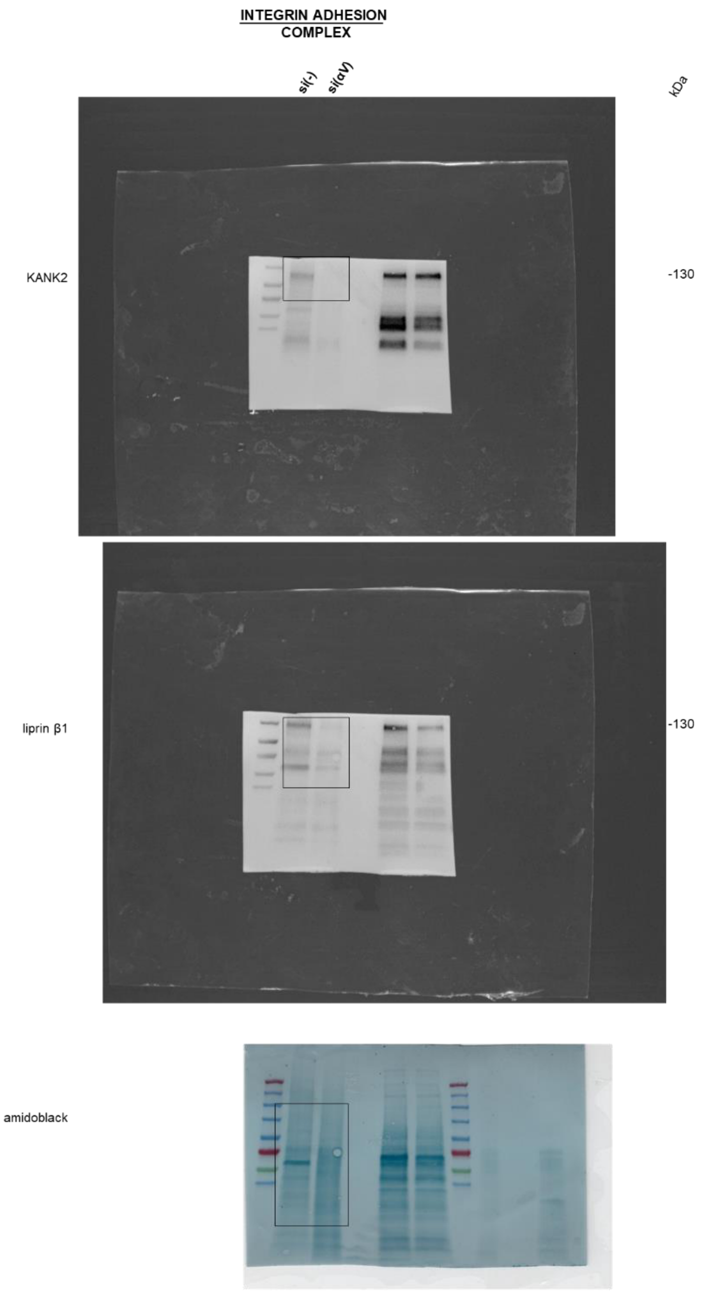

Supplementary Fig. S11 Full images of the blots in Fig. S2C. Images were obtained using Uvitec Alliance Q9 mini, which directly scanned membranes developed with ECL reagents.

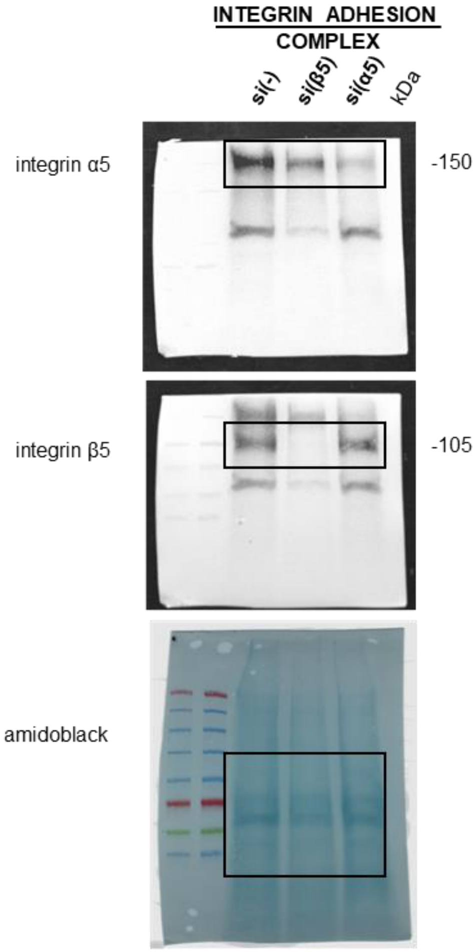

Supplementary Fig. S12 Full images of the blots in Fig. S2D. Images were obtained using Uvitec Alliance Q9 mini, which directly scanned membranes developed with ECL reagents.

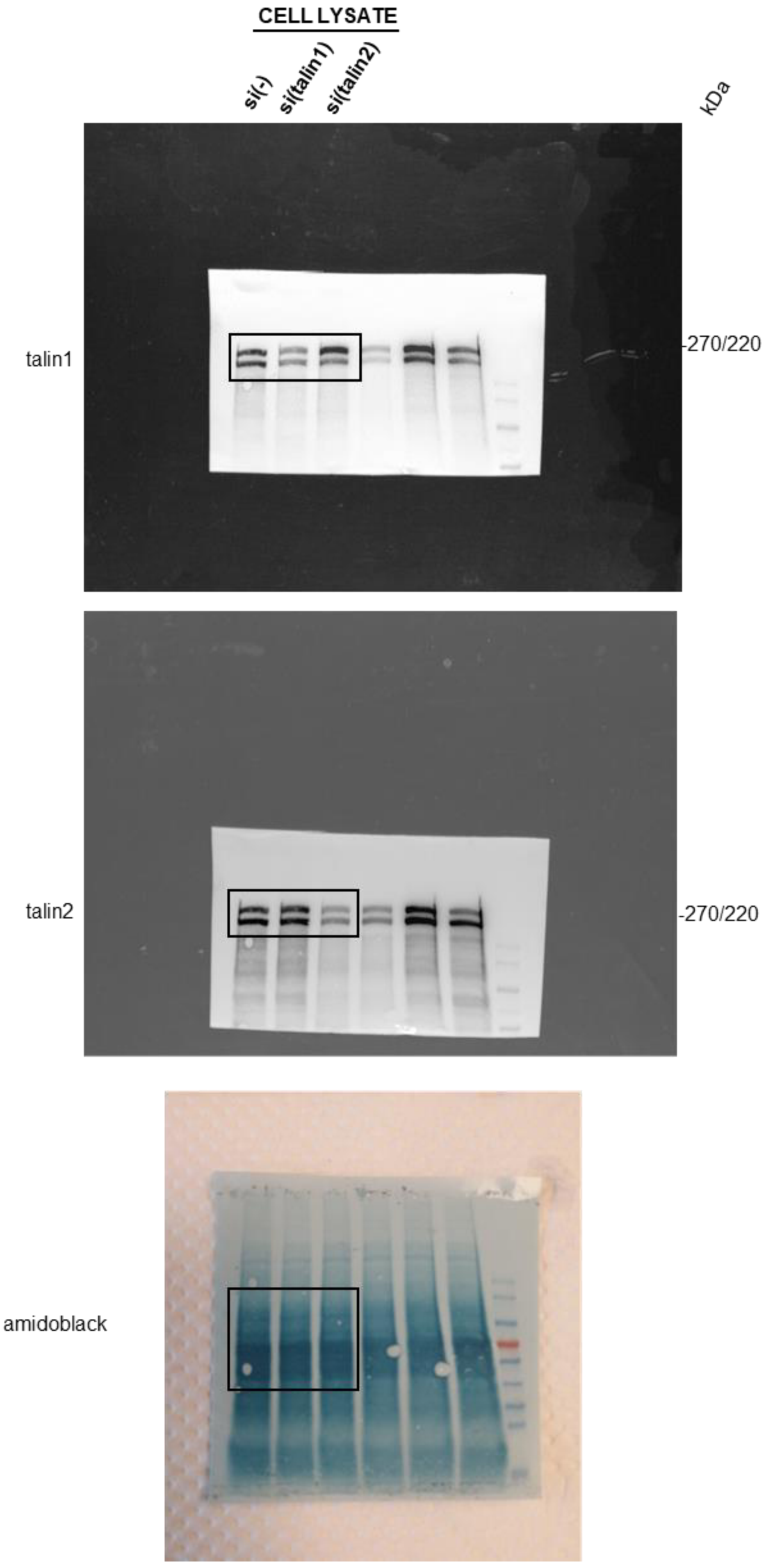

Supplementary Fig. S13 Full images of the blots in Fig. S5A. Images were obtained using Uvitec Alliance Q9 mini, which directly scanned membranes developed with ECL reagents.

Additional file 4: Supplementary Movie S1. Time-lapse live cell microscopy of RPMI-7951 cells with fluorescent EB3 (RPMI-7951-EB3 cells) transfected with control siRNA used for measurement of velocity of MT growth.

Additional file 5: Supplementary Movie S2. Time-lapse live cell microscopy of RPMI-7951 cells with fluorescent EB3 (RPMI-7951-EB3 cells) transfected with talin2-specific siRNA used for measurement of velocity of MT growth.

Additional file 6: Supplementary Movie S3. Time-lapse live cell microscopy of RPMI-7951 cells with fluorescent EB3 (RPMI-7951-EB3 cells) transfected with integrin α5-specific siRNA used for measurement of velocity of MT growth.

Additional file 7: Supplementary Movie S4. Time-lapse live cell microscopy of RPMI-7951 cells with fluorescent EB3 (RPMI-7951-EB3 cells) transfected with KANK2-specific siRNA used for measurement of velocity of MT growth.
